# Valorization of Avocado (*Persea americana*) Peel and Seed: Functional Potential for Food and Health Applications

**DOI:** 10.3390/antiox14091032

**Published:** 2025-08-22

**Authors:** Amanda Priscila Silva Nascimento, Maria Elita Martins Duarte, Ana Paula Trindade Rocha, Ana Novo Barros

**Affiliations:** 1 Academic Unit of Food Engineering, Federal University of Campina Grande, Campina Grande 58429-900, Brazil; amandapriscil@yahoo.com.br (A.P.S.N.); melitamd@gmail.com (M.E.M.D.); ana_tridade@yahoo.com.br (A.P.T.R.); 2 Centre for the Research and Technology of Agro-Environmental and Biological Sciences (CITAB), University of Trás-os-Montes e Alto Douro (UTAD), 5000-801 Vila Real, Portugal

**Keywords:** avocado by-products, *Persea americana*, peel, seed, bioactive compounds, green technologies, solid-state fermentation, functional foods, bioplastics, circular bioeconomy

## Abstract

The growing emphasis on sustainability and circular economy strategies has driven increasing interest in the valorization of agro-industrial by-products. Among these, the peel and seed of avocado (*Persea americana*), typically discarded during processing, have emerged as promising sources of bioactive compounds, particularly phenolic constituents with recognized antioxidant capacity. This review critically examines the current scientific literature on the phytochemical composition, antioxidant activity, and potential health benefits associated with avocado peel and seed. In addition, it explores recent technological advances in extraction methods and highlights the applicability of these by-products in the formulation of functional foods, nutraceuticals, and other health-related products. Challenges related to safety, bioavailability, and regulatory aspects are also discussed. By consolidating available evidence, this work supports the potential of avocado peel and seed as valuable functional ingredients and contributes to sustainable innovation in the food and health industries.

## 1. Introduction

Avocado (*Persea americana* Mill.) is a tropical fruit of Mesoamerican origin widely recognized for its nutritional richness and functional potential [1,2,3,4]. Its pulp is rich in monounsaturated fatty acids, particularly oleic acid, and also provides significant amounts of dietary fiber, fat-soluble vitamins (A, D, E, and K), carotenoids, tocopherols, and phytosterols such as β-sitosterol, all of which contribute to its well-documented health-promoting properties [5,6]. However, during industrial processing for fresh consumption or oil extraction, a considerable portion of the fruit (approximately 25–30% of its total weight) comprising mainly the peel and seed, is discarded as by-product [2,7].

In line with the growing emphasis on circular economy principles and sustainable resource use, the valorization of agro-industrial by-products has gained increasing prominence in food science and technology. Notably, avocado seed and peel, traditionally considered low-value residues, contain appreciable concentrations of phenolic acids (e.g., gallic, chlorogenic, and protocatechuic acids), flavonoids (catechin, epicatechin), tannins, and starch [3,4,8,9,10,11,12]. These phytochemicals are associated with antioxidant, antimicrobial, anti-inflammatory, and antihyperlipidemic activities, underscoring their potential as functional ingredients and natural additives [3,4,5]. Nonetheless, most compositional studies have relied on small-scale extractions under controlled laboratory conditions, often lacking standardized protocols, which hampers comparison between datasets and raises uncertainties about reproducibility at an industrial scale.

Although research and applications have largely centered on the pulp, increasing evidence highlights the potential of seed and peel extracts for incorporation into food systems, cosmeceuticals, and biodegradable packaging materials. For example, starch and polyphenols from the seed have been used to develop biodegradable films with antioxidant and barrier properties [4], while peel extracts have demonstrated protective effects against oxidative stress and apoptosis in neuronal cell models, attributed to their elevated phenolic content [5,13,14,15,16,17,18]. Yet, bioactivity evidence is still dominated by in vitro assays, with relatively few in vivo or clinical studies confirming physiological relevance and bioavailability, representing a key bottleneck for regulatory approval and product development.

From a technological perspective, innovative extraction methods, particularly ultrasound-assisted extraction and the use of green solvents (environmentally friendly solvents derived from renewable sources that reduce toxicity and environmental impact) such as natural deep eutectic solvents (NADES) [6,19,20], have enabled more sustainable and efficient recovery of phenolic compounds from avocado by-products. Despite their promise, these approaches often face scalability challenges, including energy requirements, solvent recovery, and integration into existing processing lines, which must be critically addressed before commercial adoption.

While previous reviews on avocado by-products have primarily addressed either compositional aspects or isolated applications, they have seldom integrated chemical profiling, bioactivity evidence, technological processing, and regulatory considerations into a unified valorization framework. This review distinguishes itself by (i) linking biochemical composition with demonstrated biological activities and specific technological valorization routes; (ii) emphasizing sustainable and emerging processing methods such as ultrasound-assisted extraction, NADES, solid-state fermentation, and starch modification; (iii) evaluating methodological limitations, scalability challenges, and regulatory barriers; and (iv) providing enhanced visual synthesis tools, including a comparative phytochemical composition table and an infographic mapping valorization pathways. By combining these elements, the review moves beyond summarizing the state of the art, offering a balanced assessment of current approaches while outlining strategic directions for bridging laboratory research with industrial implementation [21].

Accordingly, this work aims to provide a comprehensive and critical synthesis of current knowledge on the chemical composition, bioactivities, and technological applications of avocado peel and seed. Special emphasis is placed on their potential use as multifunctional ingredients in food, pharmaceutical, and packaging innovations, thereby contributing to sustainability and value-added product development. Framed within the context of the circular bioeconomy and sustainable food systems, this review proposes that the valorization of avocado peel and seed can be strategically optimized through the integration of biochemical characterization, green extraction technologies, and functional applications. Within this conceptual framework, we identify persistent gaps—such as lack of standardized extraction protocols, insufficient bioavailability data, and limited techno-economic analyses—that must be addressed to accelerate their transition from underutilized by-products to high-value ingredients.

## 2. Chemical Composition of Avocado By-Products

The agro-industrial processing of avocado (*Persea americana* Mill.) generates substantial quantities of solid by-product, primarily in the form of peel and seed, which together may represent up to 30% of the fruit’s total weight [3]. Historically discarded, these by-products have gained increasing scientific interest due to their complex phytochemical profiles and potential applications in food, pharmaceutical, and environmental technologies. Their chemical composition includes a wide array of polyphenols, flavonoids, phenolic acids, starch, fibers, and minerals, which collectively contribute to their multifunctional properties.

A comparative summary of representative literature values for the major phytochemical classes in avocado peel and seed is presented in Table 1. These data illustrate that the seed is particularly rich in starch, while the peel contains higher concentrations of phenolics and flavonoids, reflecting their distinct functional and technological potential. Reported values vary according to cultivar, maturity stage, and extraction method.

Reported values in Table 1 show substantial variability, reflecting differences in cultivar genetics, fruit maturity stage at harvest, agronomic conditions, and post-harvest handling [2,4,22,26]. Moreover, methodological factors—including the choice of extraction solvent, solvent-to-solid ratio, extraction time, and analytical method (e.g., Folin–Ciocalteu versus HPLC quantification for phenolics)—strongly influence the measured concentrations [6,19,20]. For example, total phenolic content in avocado peel has been reported to range from 80 to 250 mg GAE/g DW, while seed values vary from 45 to 180 mg GAE/g DW [2,4,22,26], largely due to differences in solvent polarity and sample pretreatment. Such heterogeneity limits the direct comparability of literature data and complicates the identification of reliable reference values for industrial applications. The absence of standardized extraction and quantification protocols represents a critical gap that should be addressed to improve reproducibility and facilitate cross-study benchmarking.

### 2.1. Avocado Peel

The avocado peel, traditionally regarded as a low-value agro-industrial by-product, has garnered increasing scientific interest due to its complex phytochemical composition, structural characteristics, and potential for diverse biotechnological applications [14,27]. Accounting for up to 60% of its dry weight in insoluble dietary fibers—primarily cellulose and hemicellulose—the peel not only functions as a natural protective barrier for the fruit but also exhibits excellent mechanical strength and water retention capacity. These properties render it particularly suitable for applications in the development of biodegradable materials and food packaging systems [4,6]. From a chemical perspective, the peel presents a rich and varied polyphenolic profile. Advanced chromatographic techniques such as HPLC and LC-MS have enabled the identification of numerous bioactive compounds, including chlorogenic acid, gallic acid, ferulic acid, protocatechuic acid, p-coumaric acid, and caffeic acid. Additionally, a range of flavonoids has been detected, notably rutin, quercetin derivatives, and kaempferol glycosides [3,5,28,29,30]. The total phenolic content (TPC) in avocado peel varies considerably across studies, ranging from 65 to 250 mg GAE/g dry weight, a variation influenced by factors such as cultivar, ripening stage, and extraction methodology [3,4,26]. Kouam et al. [4] conducted a comparative study of avocado varieties in Cameroon, demonstrating that the peel presented higher antioxidant capacity than the pulp or seed when assessed through the diphenylpicrylhydrazyl (DPPH), 2,2′-azino-bis(3-ethylbenzothiazoline-6-sulfonic acid) (ABTS), and ferric reducing antioxidant power (FRAP) assays. This corroborates earlier findings by Gómez-López et al. [3] in Venezuelan accessions, where chlorogenic acid and rutin predominated. Furthermore, Quintero-Espinosa et al. [5] demonstrated in human neuronal cells that phenolic-rich extracts from avocado peel could inhibit oxidative damage and apoptosis by blocking phosphorylation of Leucine-rich repeat kinase 2 (LRRK2 kinase), suggesting neuroprotective potential.

The variability in phytochemical composition among avocado cultivars has been widely reported, particularly regarding the distribution of flavonoids, phenolic acids, and antioxidant activity. Studies conducted by Gómez-López et al. [3] in Venezuela and Kouam et al. [4] in Cameroon demonstrated that both environmental and genetic factors strongly influence the concentration of bioactive compounds in avocado peel, seed, and pulp. These findings underscore the importance of cultivar selection and growing conditions in determining the functional potential of avocado by-products.

From a technological standpoint, green extraction techniques have played a pivotal role in enabling the recovery of functional phenolics from the peel. Rodríguez-Martínez et al. [6] demonstrated that natural deep eutectic solvents (NADES), when combined with ultrasound-assisted extraction (UAE), improved the selectivity and efficiency in isolating chlorogenic and ferulic acids, while reducing environmental impact compared to traditional solvents. Likewise, Salazar-López et al. [30] emphasized the importance of the peel as a source of natural antioxidants for use in functional foods and nutraceuticals, particularly when extracted via eco-friendly methods.

In addition to polyphenols, the peel contains micronutrients such as potassium, magnesium, calcium, and trace elements including iron and zinc [4,26,31]. It also harbors lipophilic antioxidants like tocopherols and carotenoids, although in lower concentrations than the pulp [1,30]. These compounds contribute to its bioactive potential in food preservation and to the development of active films and edible coatings [6,32].

The functional importance of the avocado peel extends to cardiovascular protection. Olas [28], in a comprehensive review, reported that phenolics derived from avocado peel could suppress low density lipoprotein (LDL) oxidation, modulate pro-inflammatory cytokines, and improve endothelial function in vitro and in animal models, suggesting multifaceted health benefits beyond antioxidant activity.

In summary, avocado peel represents a structurally robust and bioactive-rich biomass that remains largely underutilized despite its significant potential. Its high content of phenolic compounds, dietary fibers, and functional phytochemicals positions it as a promising raw material for the development of value-added products, including functional food ingredients, natural antioxidants, and biodegradable packaging solutions. The strategic valorization of this by-product not only enhances resource efficiency and reduces agro-industrial by-products but also aligns with circular bioeconomy principles, fostering sustainable innovation across the food, pharmaceutical, and environmental sectors.

### 2.2. Avocado Seed

The avocado seed, which accounts for approximately 13–18% of the total fruit weight, is often discarded as a by-product of industrial processing. However, [33,34] a growing body of evidence highlights its nutritional richness, polyphenolic complexity, and technological potential, which make it a highly valuable matrix for applications in food, pharmaceutical, and cosmetic industries [2,4,26,35].

From a macronutrient perspective, the seed is composed predominantly of starch, which may represent up to 70% of its dry weight, with high amylopectin content and favorable gelatinization properties [2,22,26]. These characteristics enable its use in the production of biodegradable films, edible coatings, and starch-based hydrogels for food and pharmaceutical applications [35,36].

In terms of micronutrients, the seed contains relevant concentrations of potassium, calcium, magnesium, and phosphorus, as well as trace elements like iron and zinc, contributing to its nutritional and nutraceutical potential [4,22,26]. Sánchez-Quezada et al. [26] demonstrated that mineral and polyphenolic profiles of avocado seeds are significantly affected by the ripening stage, emphasizing the need for standardization in processing conditions to ensure consistent bioactivity.

Phytochemically, the avocado seed is characterized by a diverse range of bioactive compounds, particularly phenolic acids (gallic, protocatechuic, vanillic, and ferulic acids), flavonoids (catechin, epicatechin), and tannins [2,4,5,22]. The total phenolic content (TPC) reported in the literature ranges from 45 to 180 mg GAE/g dry weight, depending on the extraction method and cultivar [3,4,22].

The antioxidant capacity of avocado seed extracts has been extensively demonstrated through DPPH, ABTS, and FRAP assays [2,4,22]. Ong et al. [22] reported that pressurized hot water extraction (PHWE) significantly improved phenolic yield and antioxidant activity, offering a green and scalable method for seed valorization. These findings are supported by Salazar-López et al. [30], who emphasized the oxidative stability and functional potential of seed extracts when incorporated into food matrices.

In terms of biological activity, Quintero-Espinosa et al. [5] demonstrated that phenolic-rich extracts from avocado seed inhibited oxidative stress and apoptosis in neuronal cell models by downregulating phosphorylation of LRRK2 kinase, suggesting neuroprotective potential. Athaydes et al. [29] further confirmed the seed’s anti-ulcer properties, showing that its oral administration in mice prevented indomethacin-induced gastric lesions, likely via antioxidant and anti-inflammatory pathways.

Recent studies have also explored advanced technological applications. Basu et al. [32] developed mesoporous silica nanoparticles decorated with galactose, loaded with avocado seed extract, for the targeted treatment of sorafenib-resistant hepatocellular carcinoma, demonstrating enhanced uptake and cytotoxicity in vitro. This represents an innovative platform for the delivery of bioactive compounds from agro-industrial by-products.

Additionally, Dabas et al. [36] proposed the use of colored avocado seed extract as a natural food colorant due to its unique anthocyanin-like pigmentation and antioxidant properties, reinforcing the multifunctionality of the seed in the food industry.

From a sustainability and economic standpoint, Charles et al. [35] highlighted that avocado seeds possess properties relevant for both food and cosmetic formulations, such as emulsion stabilization, biofilm formation, antioxidant preservation, and skin protection, opening new commercial avenues beyond food applications.

The avocado seed represents a valuable source of bioactive compounds, including phenolic acids, flavonoids, tannins, and resistant starch. These constituents have demonstrated antioxidant, antimicrobial, and anti-inflammatory properties, supporting their potential application in the development of functional foods, nutraceuticals, and natural additives. Additionally, its high content of resistant starch and polyphenols suggests prebiotic benefits and possible roles in modulating metabolic health. From a technological perspective, the seed’s physicochemical properties make it a suitable candidate for producing biodegradable films and active packaging materials. Thus, its utilization aligns with current trends in sustainable product development and the principles of circular bioeconomy.

Despite increasing scientific interest, key challenges remain. The lack of standardized methods for extracting and quantifying phenolics limits the comparability of results across studies. Furthermore, most biological activity data are based on in vitro or preclinical models, with limited validation in human studies and insufficient safety assessments over prolonged exposure. To advance the practical application of avocado seed derivatives, future research should focus on harmonizing analytical protocols, conducting well-structured clinical trials, and exploring scalable processing technologies. These efforts are essential for the safe and effective incorporation of avocado seed into food, pharmaceutical, and cosmetic formulations.

## 3. Biological Activities of Avocado Peel and Seed Extracts

Avocado by-products, especially the peel and seed, are gaining recognition as rich sources of bioactive compounds, notably phenolic compounds—including flavonoids—as well as tannins and carotenoids. These [30,37,38] phytochemicals are associated with a wide range of biological activities that support their potential application in food, pharmaceutical, and nutraceutical formulations. Among the most documented effects are antioxidant, antimicrobial, anti-inflammatory, antidiabetic, neuroprotective, and anticancer properties. These [7] effects have been demonstrated through various in vitro and in vivo models, with increasing evidence from preclinical studies that elucidate the molecular mechanisms involved—such as modulation of oxidative stress, inflammatory signaling pathways (e.g., NF-κB, COX-2), and glucose metabolism. This section presents a critical synthesis of the current scientific literature on the bioactivity of avocado peel and seed extracts, highlighting their relevance as functional ingredients for health-promoting strategies.

### 3.1. Antioxidant Activity (In Vitro and In Vivo)

The antioxidant potential of avocado peel and seed is well documented and primarily attributed to their high levels of phenolic acids (e.g., chlorogenic, gallic, protocatechuic), flavonoids (e.g., catechin, rutin), and tannins. These bioactive compounds can neutralize free radicals, chelate pro-oxidant metal ions, and modulate endogenous antioxidant enzymes [1,2,3,4].

Antioxidant capacity is commonly evaluated using in vitro assays such as 2,2-diphenyl-1-picrylhydrazyl (DPPH), 2,2′-azino-bis(3-ethylbenzothiazoline-6-sulfonic acid (ABTS), Ferric Reducing Antioxidant Power (FRAP), and Oxygen Radical Absorbance Capacity (ORAC), which measure radical scavenging activity and reducing power. Consistently, both peel and seed extracts have demonstrated high activity in these assays. For example, Kouam et al. [4] reported significantly higher DPPH and FRAP values for ethanolic extracts of peel and seed compared to pulp, correlating with total phenolic content (TPC). Similarly, Ong et al. [22] observed that pressurized hot water extraction (PHWE) of seed enhanced ABTS activity by releasing thermolabile phenolics.

Beyond chemical assays, cellular models have provided mechanistic insights. Quintero-Espinosa et al. [5] showed that avocado peel extract reduced oxidative stress in human SH-SY5Y neuronal cells exposed to paraquat/maneb by preventing apoptosis and reducing ROS (reactive oxygen species) accumulation, potentially through inhibition of LRRK2 phosphorylation, a kinase linked to Parkinson’s disease.

In vivo evidence also supports strong antioxidant effects. Athaydes et al. [29] found that oral administration of seed extract to mice prevented indomethacin-induced gastric mucosal injury, reduced malondialdehyde (MDA) levels, and enhanced superoxide dismutase (SOD) and catalase (CAT) activities. Similarly, Salazar-López et al. [30] demonstrated that phenolic-rich peel extracts improved oxidative stability in food matrices such as meat emulsions and oils, reinforcing their role as natural preservatives.

Taken together, these findings confirm that avocado peel and seed possess robust antioxidant capacity through both direct radical scavenging and modulation of enzymatic defense mechanisms. The methodological details and general evidence for antioxidant activity are summarized in this section; in subsequent sections, antioxidant effects are referenced here and discussed only when directly relevant to a specific biological or technological context.

Reported antioxidant capacities for avocado peel and seed extracts show considerable variability across studies, which can be attributed to multiple factors, including differences in extraction methods, solvent polarity, extraction time and temperature, as well as cultivar-specific phytochemical profiles and maturity stage at harvest [2,4,22,26,39]. Moreover, the choice of antioxidant assay—such as DPPH, ABTS, FRAP, or ORAC—and differences in assay standardization significantly affect the reported values [4,22]. For example, while some studies report DPPH radical scavenging activity exceeding 90% for ethanolic peel extracts [4], others have observed substantially lower values for aqueous or less polar extractions [22,39]. This methodological heterogeneity complicates direct cross-study comparisons and highlights the need for standardized extraction protocols and harmonized bioactivity assays to improve reproducibility and enable robust benchmarking across research groups.

### 3.2. Antimicrobial Activity (Against Salmonella, Listeria, and Escherichia coli)

The increasing global concern over antimicrobial resistance and foodborne illnesses has intensified interest in natural compounds capable of controlling pathogenic bacteria. In this regard, avocado by-products, particularly the peel and seed, have demonstrated promising antimicrobial activity due to their rich content of phenolic acids, flavonoids, condensed tannins, and saponins, which disrupt bacterial membrane integrity, interfere with protein synthesis, and induce oxidative stress in microbial cells [1,2,4].

Multiple studies have shown that ethanolic and aqueous extracts of avocado peel and seed exert inhibitory effects against both Gram-positive and Gram-negative bacteria, including *Escherichia coli*, *Salmonella enterica*, and *Listeria monocytogenes* [2,4,35]. In a comprehensive review, Charles et al. [35] reported that avocado seed extract inhibited *E. coli* and *Staphylococcus aureus* at concentrations as low as 2 mg/mL, mainly through mechanisms involving cell membrane disruption and increased permeability.

A particularly relevant study by Trujillo-Mayol et al. [40] demonstrated that fractionation and enzymatic hydrolysis of avocado peel significantly enhanced its antibacterial activity. Hydrolyzed avocado peel extracts (HAPE) exhibited over 80% biofilm inhibition of *Listeria monocytogenes* after 12 h and showed effective growth suppression of *E. coli* and *Salmonella enterica*. The increased efficacy was attributed to the release of low-molecular-weight phenolics such as gallic acid and catechin, with improved diffusion and cell penetration.

Salazar-López et al. [30] further confirmed that green-extracted peel compounds produced clear zones of inhibition against *L. monocytogenes* and *S. typhimurium*, suggesting potential for use as natural preservatives in processed and ready-to-eat foods. These findings are particularly relevant for the development of active packaging systems and natural antimicrobial additives.

In another study, Ong et al. [22] incorporated avocado seed extract into starch-based biodegradable films, demonstrating dose-dependent inhibition of *E. coli* and *S. aureus*, thereby validating its application in antimicrobial food packaging.

The activity against biofilm-forming pathogens is especially significant, as strains such as *E. coli* and *Salmonella* spp. are known to persist on food-contact surfaces. The presence of bioactive compounds like protocatechuic acid, chlorogenic acid, and catechins has been associated with membrane destabilization, inhibition of quorum sensing, and interference with the formation of extracellular polymeric substances [1,2,40].

Taken together, the evidence supports that avocado peel and seed extracts, especially when processed through advanced techniques such as hydrolysis, pressurized hot water extraction, or ultrasound-assisted methods, offer effective antimicrobial activity against critical foodborne pathogens. These by-products represent sustainable alternatives for application in food preservation, bioactive packaging, and natural sanitizing systems.

### 3.3. Anti-Inflammatory and Anticancer Activities

Avocado by-products, particularly the peel and seed, are increasingly recognized as promising sources of bioactive compounds with clinically relevant anti-inflammatory and anticancer activities. These effects have been associated with their rich content of polyphenols, flavonoids, procyanidins, and tannins, which are known to modulate molecular pathways involved in inflammation and tumor development [28,41].

Several in vitro studies have demonstrated that avocado seed and peel extracts are capable of modulating key inflammatory mediators. Kupnik et al. [41] showed that enzymatically extracted compounds from avocado seed significantly inhibited the activity of lipoxygenase and cyclooxygenase (COX) enzymes while suppressing nitric oxide (NO) production in LPS-stimulated RAW 264.7 macrophages. These results were supported by reductions in the expression of inducible nitric oxide synthase (iNOS) and in the release of pro-inflammatory cytokines such as tumor necrosis factor-alpha (TNF-α) and interleukin-6 (IL-6).

Supporting this evidence, BaiBerrios-Henríquez et al. [42] isolated epicatechin adducts derived from procyanidins in avocado peel using centrifugal partition chromatography. These purified compounds were able to inhibit TNF-α and IL-1β production in THP-1 monocytes and demonstrated selective cytotoxicity toward HeLa and A549 cancer cell lines, with IC_50_ values ranging from 70 to 90 µM, alongside marked antioxidant capacity.

These in vitro observations have also been validated in animal models. Skenderidis et al. [43] assessed the anti-inflammatory potential of aqueous extracts of avocado peel and seed obtained via vacuum microwave-assisted extraction. In a murine model of carrageenan-induced paw edema, treatment with the extract significantly reduced inflammation, lowered plasma IL-6 concentrations, and downregulated COX-2 and iNOS expression in inflamed tissues. The findings indicate that avocado by-products not only exert antioxidant effects but also modulate inflammatory gene expression in vivo.

Importantly, the anticancer effects of these by-products have also been well documented. Kupnik et al. [41] demonstrated that seed-derived bioactives induced apoptosis in HT-29 and MCF-7 cells by promoting caspase-3 activation, poly-(ADP-ribose) polymerase (PARP) cleavage, and G0/G1 cell cycle arrest. The effect was dose-dependent and was further enhanced by co-treatment with low concentrations of doxorubicin, suggesting a synergistic interaction between avocado polyphenols and conventional chemotherapy agents.

Further evidence comes from in vivo cancer models. BaiBerrios-Henríquez et al. [42] reported that administration of purified avocado peel epicatechin adducts led to a ~40% reduction in tumor volume in xenograft models of lung cancer, with no evidence of systemic toxicity, underscoring their potential for selective tumor inhibition.

Complementing these mechanistic and preclinical insights, Donoso et al. [44] demonstrated that extracts from Hass avocado peel exhibited potent antioxidant and antimicrobial activity against phytopathogenic fungi such as *Aspergillus niger*, *Verticillium theobromae*, and *Colletotrichum musae*. Although focused on plant pathogens, these findings reinforce the multifunctionality of avocado peel phenolics and suggest broader biological potential.

Finally, Olas [28] reviewed a range of experimental data supporting the role of avocado peel and seed extracts in reducing systemic inflammation and oxidative stress. These effects include inhibition of NF-κB signaling, modulation of lipid metabolism, and attenuation of chronic inflammation, which collectively contribute not only to cardiometabolic protection but also to the suppression of tumor-promoting microenvironments.

Taken together, these findings highlight the dual role of avocado peel and seed as potent anti-inflammatory agents and as natural sources of cytotoxic compounds with relevance for cancer prevention and therapy. Their multimodal mechanisms of action, combined with low toxicity and compatibility with green extraction techniques, support their use in the development of functional foods, nutraceuticals, and adjunctive therapeutic strategies for inflammatory and neoplastic diseases [35].

Recent advances indicate that nanoencapsulation (a technique in which bioactive compounds are enclosed within nanometer-scale carriers to protect them from degradation, enhance stability, improve bioavailability, and allow controlled release) may further enhance the functional potential of avocado peel and seed bioactives compared with crude extracts. Encapsulation in biopolymeric carriers, such as chitosan, offers protection against oxidative degradation and adverse gastrointestinal conditions, improving solubility and intestinal absorption. Sahyon et al. [45] demonstrated that chitosan nanoparticle-encapsulated avocado peel extract retained higher antioxidant activity and exhibited superior antitumor efficacy in a urethane-induced lung cancer mouse model compared with the non-encapsulated extract. These improvements were attributed to increased bioavailability and controlled release of active compounds in target tissues. However, despite promising preclinical findings, no robust human clinical trials have yet confirmed these benefits. Well-designed studies addressing bioavailability, pharmacokinetics, and safety will be essential to validate encapsulation as a strategy for developing effective functional foods, nutraceuticals, and therapeutic formulations.

### 3.4. Neuroprotective and Antidiabetic Potential

By-products from avocado processing, particularly the seed and peel, have shown promising bioactivity in the prevention and modulation of neurodegenerative and metabolic disorders. Their rich phytochemical composition, including flavonoids, procyanidins, chlorogenic acid, catechins, and other phenolic compounds, plays a central role in these effects through mechanisms such as antioxidant action, enzyme inhibition, and modulation of intracellular signaling pathways (see Section 3.1 for a detailed discussion of antioxidant mechanisms).

From a neurological perspective, inhibition of acetylcholinesterase (AChE) and butyrylcholinesterase (BChE) is considered a relevant target in the management of neurodegenerative conditions such as Alzheimer’s disease. Del-Castillo-Llamosas et al. [20] reported that agri-food by-products, including avocado peel and seed, exhibit both cholinesterase inhibitory activity and strong antioxidant potential. Supporting these findings, da Silva et al. [16] demonstrated that ethanolic extracts from avocado peel and seed inhibited AChE activity by over 80% and provided neuroprotective effects in *Drosophila melanogaster*, improving survival and locomotor function under oxidative stress. These effects were attributed to enzymatic inhibition as well as attenuation of oxidative damage in neuronal cells.

Regarding metabolic health, the antidiabetic potential of avocado seed has been consistently reported in preclinical studies. Ojo et al. [39] found that aqueous seed extract administration in diabetic rats significantly reduced blood glucose, LDL-cholesterol, triglycerides, and pro-inflammatory cytokines such as TNF-α and IL-6, effects linked to enhanced insulin secretion, upregulation of the Phosphoinositide 3-kinase/protein kinase B (PI3K/Akt) pathway, and increased expression of anti-apoptotic proteins such as Bcl-2. Similarly, Uchenna et al. [46] reported that dietary supplementation with avocado seed powder improved glycemic control and lipid metabolism in hypertensive rats while preserving pancreatic histoarchitecture. A systematic review by Agunloye et al. [47] integrating data from 45 preclinical studies confirmed that avocado extracts consistently improved glycemic indices and mitigated oxidative stress-related damage.

Although these findings consistently point to neuroprotective and antidiabetic potential, interpretation is complicated by substantial heterogeneity in experimental design. Differences in plant material preparation (fresh versus dried), extraction methods (ethanolic, aqueous, hydroalcoholic), solvent polarity, and concentration of active constituents all contribute to variability in reported outcomes [2,4,22,26,39]. In animal models, doses, treatment durations, and administration routes vary widely, making it difficult to compare effect sizes or establish optimal therapeutic ranges [39,46,47]. Moreover, most evidence to date comes from in vitro assays or short-term preclinical studies, with few well-controlled human clinical trials. The lack of standardized dosing protocols, combined with scarce data on bioavailability, pharmacokinetics, and long-term safety, limits translational potential. Overcoming these challenges will require standardized experimental designs, harmonized analytical methods, advanced multi-omics approaches to elucidate mechanisms, and robust clinical trials to validate efficacy and safety in humans.

### 3.5. Summary of Bioactivities of Avocado Peel and Seed

To provide an integrated overview of the biological activities described in the preceding sections, Table 2 summarizes the main findings reported for avocado peel and seed extracts. The table compiles the bioactivity type, plant part studied, experimental model, key outcomes, and references. This synthesis allows direct comparison between different biological endpoints and reinforces the multifunctionality of avocado by-products as valuable sources of bioactive compounds for potential nutraceutical, pharmaceutical, and functional food applications.

## 4. Technological Potential and Industrial Applications

The valorization of agro-industrial by-products has emerged as a strategic priority in sustainable food systems in alignment with the principles of the circular economy and clean technologies. Among these by-products, the peel and seed of avocado (*Persea americana* Mill.) are particularly rich in bioactive compounds and remain significantly underutilized [27]. These by-products are processed using technologies such as encapsulation, lipid removal, green extraction, and micronization, with potential applications in cosmetics after stabilization.

Recent advances in food engineering, materials science, and biotechnology have enabled the transformation of these by-products into high-value-added ingredients for use in food, pharmaceutical, cosmetic, and packaging applications. Such innovations contribute not only to reducing agro-industrial by-products but also to promoting sustainability and adding value across the avocado supply chain [27].

### 4.1. Encapsulation of Bioactive Compounds from Peel and Seed

Although the peel and seed of avocado are recognized for their rich phytochemical profiles, the practical application of these bioactives in functional products remains limited due to their intrinsic chemical instability and low bioavailability. Exposure to light, heat, oxygen, and pH fluctuations can degrade sensitive compounds such as phenolic acids and flavonoids, thereby reducing their efficacy in food and therapeutic systems. To overcome these barriers, several encapsulation technologies have been developed to protect these compounds, control their release, and enhance their stability within diverse matrices [27].

Féliz-Jiménez and Sanchez-Rosario [27] highlighted the growing importance of encapsulation techniques employing natural biopolymers such as chitosan, cyclodextrins, and maltodextrin to protect bioactive extracts from avocado by-products. These systems have demonstrated effectiveness in preserving antioxidant and anti-inflammatory properties, while also improving their suitability for incorporation into food and nutraceutical products.

Sahyon et al. [45] successfully encapsulated avocado peel extract into chitosan nanoparticles via nanoprecipitation. The resulting particles showed mean diameters of 166–305 nm, a polydispersity index of 0.19–0.26, and encapsulation efficiency exceeding 80%. In vitro studies revealed significant antioxidant and pro-apoptotic activity in leukemia cell lines. In a urethane-induced lung cancer mouse model, the nanoparticles also demonstrated tumor suppression, with increased expression of apoptotic markers (p53 and Bax) and downregulation of NF-κB p65, confirming their therapeutic potential.

In parallel, Nguyen et al. [48] applied refractance window drying with maltodextrin to protect the phenolic content of avocado pulp. Although the study focused on pulp, the authors suggested that the method could also be applied to peel and seed extracts due to their similar thermal behavior and polyphenolic composition. The encapsulation process preserved more than 85% of total phenolic compounds and maintained antioxidant capacity, indicating promising applicability for stabilizing bioactives from avocado by-products.

Taken together, these studies highlight that encapsulation strategies not only enhance the functional value and technological usability of avocado-derived bioactives but also facilitate their broader integration into sustainable industrial applications.

### 4.2. Fermentation of Avocado By-Products for Bioingredient Production

Fermentation has emerged as a powerful biotechnological tool for enhancing the value of agro-industrial by-products, including avocado (*Persea americana* Mill.) by-products such as the seed and peel. These materials, rich in phenolics, fibers, and carbohydrates, offer an excellent substrate for microbial transformation aimed at producing functional bioingredients.

Recent studies have demonstrated the potential of solid-state fermentation (SSF) as an effective method to improve the nutritional and functional properties of avocado seeds. Villasante et al. [49] reported that fermentation with *Aspergillus oryzae* and *A. awamori* increased protein content and improved antioxidant capacity (see Section 3.1 for methodological details) while also generating volatile compounds associated with enhanced sensory attributes. This microbial bioprocessing promoted the release of bound bioactives and enriched the nutritional profile of the fermented material.

Zhao et al. [50] combined fermentation with *Lactobacillus plantarum* and roasting of Hass avocado seeds, resulting in a 40% increase in total phenolic content and improved antioxidant potential. The fermented extracts also showed significant cytotoxicity against HepG2, MCF-7, and MDA-MB-231 cancer cell lines, supporting their potential use in functional foods or nutraceutical formulations targeting oxidative stress and tumor suppression.

Another promising application involves the use of fermented avocado seed extracts as natural inhibitors of enzymatic browning. Yepes Betancur et al. [51] demonstrated that fermented extracts effectively inhibited polyphenol oxidase (PPO) activity in fresh-cut avocado, apple, and banana pulps, positioning them as a clean-label alternative to synthetic anti-browning agents in minimally processed fruit products.

Fermentation has also been explored for biofuel production. Caballero Sánchez et al. [52] conducted a pilot-scale study using dilute acid hydrolysis followed by yeast fermentation, achieving an ethanol yield of 0.44 g/g of glucose and a final concentration exceeding 50 g/L. These results support the feasibility of converting avocado seed starch into second-generation bioethanol, contributing to sustainable energy solutions.

Collectively, these studies highlight the versatility of fermentation as a tool for the functionalization of avocado by-products. Whether through enhancement of bioactivity, enzyme inhibition, flavor development, or renewable energy generation, fermentation remains a central pillar in the circular bioeconomy approach to avocado by-product valorization.

### 4.3. Advances in Food Engineering for the Transformation of Avocado By-Products into Premium Ingredients

Food engineering has played a crucial role in developing sustainable technologies for the transformation of agro-industrial by-products into high-value products. Among these, the peel and seed of avocado (*Persea americana* Mill.) are rich in fibers, starch, phenolic compounds, and antioxidants, making them ideal candidates for conversion into functional ingredients with technological applications in food, cosmetics, and nutraceuticals [36,53].

Several emerging techniques have demonstrated promising outcomes. Figueroa et al. [23] used microwave-assisted extraction to recover phenolic compounds from avocado peel, obtaining extracts with high antioxidant capacity (see Section 3.1 for methodological details) and the ability to inhibit matrix metalloproteinases—enzymes associated with tissue degradation. These properties suggest potential applications in functional foods and natural food preservation.

Velderrain-Rodríguez et al. [54] evaluated phenolic-rich extracts from avocado by-products, reporting not only significant antioxidant effects but also antiproliferative activity against colon and breast cancer cell lines. These findings support the use of avocado by-products as bioactive components in health-promoting food formulations.

In terms of structural applications, the seed has been explored as a source of starch and flour with favorable physicochemical and rheological characteristics. De Dios-Avila et al. [53] demonstrated that starch extracted from both Hass and Landrace avocado cultivars presented high thermal stability and low retrogradation, making it suitable for food and pharmaceutical products.

Furthermore, Silva et al. [24] characterized native and acetylated starches from avocado seeds, noting improvements in swelling power, solubility, and paste clarity after modification—features relevant for sauces, bakery fillings, and frozen food applications. Bet et al. [55] further showed that lactic acid-modified starch exhibited enhanced thermal stability and film-forming capacity, reinforcing its utility in biodegradable packaging.

Food engineering has also enabled the development of microbial culture media from avocado seed by-products. Camacho et al. [56] formulated a low-cost growth substrate for lactic acid bacteria using hydrolysates of avocado seed, which sustained bacterial viability and acid production, offering an innovative application for probiotic or fermentative systems.

The phytochemical richness of these by-products has also been documented by Dabas et al. [36], who reviewed their bioactive profiles, emphasizing potential as functional food ingredients. Additionally, recent studies have highlighted the technological relevance of avocado oil and its processing by-products. Cervantes Paz and Yahia [57] provided a comprehensive overview of extraction techniques, fatty acid composition, and bioactive constituents of avocado oil, emphasizing its role in the development of functional lipid matrices, emulsified systems, and nutraceutical products. The authors noted the high content of oleic acid, tocopherols, phytosterols, and chlorophylls, which confer both oxidative stability and health-promoting properties to the oil.

Krumreich et al. [25] confirmed that the extraction method significantly influences the physicochemical and phytochemical attributes of avocado oil. Oils obtained through cold pressing retained greater levels of phenolic compounds and exhibited higher antioxidant activity compared to those extracted via solvent methods. These findings are crucial for the development of clean-label, high-value lipid systems, particularly for use in food emulsions, lipid structuring, and microencapsulation technologies.

Together, these advances illustrate that avocado by-products, including oil-rich fractions and residual press cakes, can be strategically transformed into premium functional ingredients through the application of food engineering and green processing technologies [36,53,57]. This not only enhances product functionality and nutritional quality but also supports sustainable innovation within circular economy frameworks, reducing by-products and adding value to the agro-industrial chain.

### 4.4. Fermentation of Avocado By-Products for Bioenergy and Technological

Fermentation has gained prominence as a sustainable and efficient strategy for the valorization of agro-industrial by-products. In the case of avocado (*Persea americana* Mill.), the peel and seed provide suitable substrates for microbial biotransformation due to their composition rich in fibers, starches, and phenolic compounds. These fermentation-based approaches contribute to by-product mitigation while enabling the development of functional ingredients with enhanced bioactivity and broader applicability [1,27].

Solid-state fermentation (SSF) is particularly suited for lignocellulosic matrices such as avocado peel and seed. Villasante et al. [49] reported that fermentation of avocado seed with *Aspergillus oryzae* and *A. awamori* increased protein content by 27% and improved antioxidant capacity (see Section 3.1 for methodological details) while generating bioactive volatiles with potential applications in nutraceuticals and functional foods.

In a targeted study, Zhao et al. [50] evaluated the antioxidant and anticancer properties of fermented avocado seed extracts, followed by a roasting step. The extracts showed improved free radical scavenging capacity and induced cytotoxic effects on HepG2, MCF-7, and MDA-MB-231 cancer cell lines, reinforcing their potential for nutraceutical applications.

Beyond bioactivity, fermented extracts also present promising technological applications. Yepes Betancur et al. [51] demonstrated that fermented avocado seed inhibited polyphenol oxidase (PPO) activity in fresh-cut fruits, reducing enzymatic browning in avocado, apple, and banana pulps. The extract, when applied at 60% (*v*/*v*), significantly delayed browning, offering a clean-label solution for post-harvest preservation.

Fermentation can also support energy applications. Caballero Sánchez et al. [52] reported the pilot-scale production of second-generation bioethanol using avocado seed starch. The process involved acid hydrolysis followed by yeast fermentation, resulting in an ethanol yield of 0.44 g/g of glucose and final concentrations exceeding 50 g/L. These findings support the feasibility of avocado by-products as a feedstock for biofuel generation.

Collectively, these studies highlight the versatility of fermentation as a biotechnological route for the valorization of avocado peel and seed. Whether through enhancement of bioactivity, control of enzymatic browning, or bioethanol production, fermentation platforms open new possibilities for sustainable use of these agro-industrial by-products.

### 4.5. Biodegradable Packaging from Avocado By-Products

The growing volume of agro-industrial by-products from avocado processing—particularly peel and seed by-products—has prompted increased research into sustainable materials for biodegradable packaging. These by-products provide a renewable feedstock for eco-friendly packaging solutions, and their valorization aligns with circular economy principles and the global demand for alternatives to petroleum-based plastics.

Recent advances have demonstrated the feasibility of using avocado seed starch in biopolymer film development. Muñoz-Gimena et al. [58] formulated biodegradable films by blending avocado seed starch with glycerol and reinforcing them with nanocrystals. The resulting films exhibited improved tensile strength, decreased water vapor permeability, and notable antioxidant capacity (see Section 3.1 for methodological details)—an essential feature for active food packaging systems. Impressively, these films fully degraded under composting conditions within 48 h, demonstrating their environmental compatibility.

Similarly, avocado peel fibers have been explored as a viable alternative to synthetic polymers. In a study by Janaswamy and Ahmed [59], cellulose extracted from avocado peel was used to produce calcium-ion-stabilized films. These films demonstrated high transparency, excellent moisture resistance, and tensile strength comparable to polyethylene, in addition to complete biodegradation in moist soil within 30 days.

Furthermore, Dewi et al. [60] employed response surface methodology (RSM) to optimize biodegradable plastics based on avocado seed starch and polylactic acid (PLA). The resulting films combined biodegradability with good mechanical performance, suggesting potential for replacing conventional plastics in short-life packaging applications. Their compatibility with industrial composting and lower environmental impact further reinforces the relevance of starch–PLA composites.

Taken together, these studies highlight the potential of avocado peel and seed by-products as renewable sources for high-performance, eco-friendly packaging materials [59,60]. By integrating approaches from green chemistry, nanotechnology, and polymer science [35,57], these by-products can be effectively transformed into sustainable packaging solutions. Such innovations not only mitigate environmental burdens associated with synthetic plastics but also enhance the economic and environmental sustainability of the avocado processing chain [35,57].

However, despite the technical feasibility demonstrated in laboratory and pilot-scale studies, industrial implementation faces several critical challenges. Most processing strategies, including green extraction, fermentation, starch modification, and biopolymer development, remain at early-scale development with limited validation under industrial conditions [6,19,20,24,49,52,57]. Comprehensive techno-economic assessments and life-cycle analyses are scarce, hindering accurate evaluation of cost-effectiveness and environmental impact. Regulatory approvals, such as GRAS status in the United States or equivalent clearances in other regions, require robust toxicological data, compositional standardization, and consistent bioactive profiles, which are still lacking. In the case of biodegradable packaging, additional optimization is needed to improve mechanical performance, ensure stability during storage, and comply with industrial composting standards. Addressing these technological, regulatory, and economic barriers will be essential for translating promising laboratory results into commercially viable, sustainable products.

Beyond biodegradable packaging, similar constraints apply to other promising applications described in this review, such as nutraceutical formulations and natural food additives. While pilot studies have demonstrated technical feasibility in incorporating avocado by-product extracts into functional foods [30] and active packaging systems [58,60], no large-scale commercial implementations have yet been reported [35,57]. This reflects not only technical and regulatory challenges [35,57] but also the need for robust market analyses, cost–benefit evaluations, and standardized processing protocols to ensure reproducibility and consumer safety [25,57]. Without addressing these barriers, the transition from research-driven innovation to sustainable industrial adoption will remain limited.

At present, there are no specific regulatory approvals, such as Generally Recognized As Safe (GRAS) status in the United States or favorable European Food Safety Authority (EFSA) opinions in Europe, for the use of avocado peel or seed extracts in commercial foods or health products [35,57]. While avocado oil derived from the pulp has regulatory acceptance in some jurisdictions [57], by-products such as peel and seed have not yet been formally recognized as approved food ingredients. Achieving regulatory compliance will require robust toxicological data, standardization of extraction and processing methods, and submission of comprehensive safety and efficacy dossiers to the competent authorities, including Food and Drug Administration (FDA), EFSA, and Brazilian Health Regulatory Agency (ANVISA) [25,35,57].

Considerations of safety and potential antinutritional effects should be carefully considered before avocado peel and seed extracts are adopted on a large scale in foods, nutraceuticals, or pharmaceutical products. Avocado seeds naturally contain small amounts of cyanogenic glycosides and condensed tannins, which, in unprocessed form and at high intake levels, may exert antinutritional effects [1,2]. Evidence suggests that these compounds are largely reduced or eliminated through appropriate processing steps such as drying, extraction, and purification [3,4]. At present, most safety data originate from in vitro experiments or animal studies, often employing doses far exceeding those relevant for human consumption. Short-term preclinical studies have not reported major adverse effects at tested levels [5,6]; however, robust long-term human clinical trials remain absent. Comprehensive toxicological evaluation, including acute, subchronic, and chronic toxicity studies as well as assessments of potential interactions with other bioactive compounds, will be essential to meet regulatory requirements (e.g., GRAS in the United States, EFSA in Europe, ANVISA in Brazil) and to ensure consumer safety.

## 5. Conclusions and Future Perspectives

The growing demand for sustainable and circular models in the agri-food sector has prompted a critical reassessment of agro-industrial by-products, with avocado peel and seed emerging as particularly valuable raw materials. Once regarded as by-products, these by-products are now recognized for their rich and diverse composition, including phenolic compounds, dietary fibers, lipids, and starches, which support a broad range of potential applications in the food, nutraceutical, pharmaceutical, and environmental industries.

Advances in green extraction methods, such as ultrasound-assisted extraction, microwave-assisted techniques, and natural deep eutectic solvents (NADES), have markedly improved the selective recovery of bioactive compounds from these matrices. In parallel, innovations in fermentation, nanoencapsulation, and biopolymer engineering have expanded the scope of applications and enabled the development of active packaging systems, antioxidant-rich ingredients, edible films, and biocompatible materials.

These integrated and multidisciplinary approaches link food technology, biotechnology, and materials science and demonstrate the potential of avocado peel and seed to be transformed into high-value components, contributing to by-product reduction and improved resource efficiency. However, critical challenges remain. Standardization of extraction and analytical protocols is urgently needed to enable reproducibility and cross-study comparability. The bioaccessibility (fraction of a compound released from the food matrix during digestion and available for intestinal absorption), bioavailability, and long-term safety of derived compounds require thorough investigation, and robust clinical trials are lacking. While scientific and technological advances clearly support the valorization of avocado peel and seed, regulatory and market constraints still limit their industrial adoption. The absence of formal safety status (e.g., GRAS designation) for many extracts, variability in extraction outcomes, and lack of internationally harmonized standards for processing and quality control create significant bottlenecks. Additionally, industrial-scale feasibility must be demonstrated through robust techno-economic evaluations and life-cycle assessments.

To accelerate real-world implementation, future research should prioritize the following:

1. Standardization of processing and analytical protocols—Harmonize extraction, purification, and quantification methods to ensure reproducibility and cross-study comparability;
2. Bioavailability and safety assessment—Conduct in vivo studies, including long-term toxicological evaluations and well-designed human clinical trials, to establish efficacy, safety, and regulatory compliance;
3. Industrial scalability and process optimization—Demonstrate the feasibility of scaling up green extraction, fermentation, and biopolymer development through pilot-to-industrial-scale studies;
4. Regulatory approval pathways—Develop compositional standards and toxicological dossiers to support GRAS status (United States), EFSA approval (Europe), and ANVISA registration (Brazil) for food and nutraceutical use;
5. Techno-economic and environmental evaluation—Perform robust cost–benefit analyses and life-cycle assessments to confirm commercial viability and sustainability.

Addressing these priorities will be pivotal to unlocking the full potential of avocado by-products as safe, high-value ingredients for food, nutraceutical, pharmaceutical, and packaging applications. Their successful valorization will strengthen circular bioeconomy models, foster innovation in functional food systems, and promote more sustainable and resilient value chains.

## Figures and Tables

**Table 1:** Comparative phytochemical composition of avocado (*Persea americana* Mill.) peel and seed reported in the literature. Values expressed on a dry weight (DW) basis; ranges reflect differences in cultivar, maturity stage, and extraction method.

PHYTOCHEMICAL CLASS	COMPOUND EXAMPLES	PEEL (MG/G DW)	SEED (MG/G DW)	EXTRACTION YIELD
**TOTAL PHENOLIC CONTENT (TPC)**	Gallic, chlorogenic, protocatechuic acids	80–250	45–180	
**FLAVONOIDS**	Catechin, epicatechin, rutin	10–35	8–25	Peel: up to 95 mg GAE/g (NADES) [22]; Seed: up to 85 mg GAE/g (PHWE) [20]; Peel: ~30 mg/g (MAE) [23].
**TANNINS**	Condensed tannins, procyanidins	15–40	20–55	n.i. [2,3,4]
**DIETARY FIBER**	Insoluble and soluble	350–450	300–420	n.i. [3,4,12]
**STARCH**	Amylose, amylopectin	<5	550–700	Seed starch extraction yield: 60–65% DW [24]
**LIPIDS**	Oleic, linoleic, palmitic acids	20–45	15–30	Peel oil yield: ~3–5% DW; Seed oil yield: ~2–4% DW [25]
**MINERALS**	K, Ca, Mg, Fe, Zn	25–45 (total minerals)	30–55 (total minerals)	n.i. [4,22,26]

Abbreviations: TPC = total phenolic content; DW = dry weight; GAE = gallic acid equivalents; MAE = microwave-assisted extraction; NADES = natural deep eutectic solvents; PHWE = pressurized hot water extraction; n.i. = not informed.

**Table 2:** Summary of key bioactivities of avocado peel and seed extracts reported in the literature.

BIOACTIVITY	PLANT PART	STUDY MODEL	MAIN FINDINGS	REFERENCES
**ANTIOXIDANT**	Peel and Seed	In vitro (DPPH, ABTS, FRAP, ORAC)	High radical scavenging activity correlated with total phenolic content; PHWE enhanced yield of thermolabile phenolics.	[4,22,30]
**ANTI-INFLAMMATORY**	Seed	In vitro (RAW 264.7 macrophages)	Inhibition of LOX and COX enzymes; suppression of NO production; reduced TNF-α and IL-6.	[41]
**ANTI-INFLAMMATORY**	Peel	In vivo (murine carrageenan-induced paw edema)	Reduced edema, lowered IL-6, downregulated COX-2, and iNOS in inflamed tissues.	[43]
**ANTICANCER**	Seed	In vitro (HT-29, MCF-7 cells)	Induced apoptosis, PARP cleavage, G0/G1 arrest; synergistic with low-dose doxorubicin.	[41]
**ANTICANCER**	Peel	In vivo (lung cancer xenograft)	Epicatechin adducts reduced tumor volume by ~40% without systemic toxicity.	[42]
**NEUROPROTECTIVE**	Peel and Seed	In vitro (SH-SY5Y, *Drosophila* model)	AChE and BChE inhibition; protection against oxidative stress; improved survival and locomotor function.	[16,20]
**ANTIDIABETIC**	Seed	In vivo (diabetic rat models)	Reduced glucose, LDL-C, triglycerides, TNF-α, IL-6; enhanced insulin secretion and PI3K/Akt activation.	[39,46,47]
**ANTIMICROBIAL**	Peel	In vitro	Inhibition of *A. niger*, *V. theobromae*, and *C. musae*; potential as a natural antifungal agent.	[44]

Abbreviations: DPPH = 2,2-diphenyl-1-picrylhydrazyl; ABTS = 2,2′-azino-bis(3-ethylbenzothiazoline-6-sulfonic acid); FRAP = Ferric Reducing Antioxidant Power; ORAC = Oxygen Radical Absorbance Capacity; NO = nitric oxide; TNF-α = tumor necrosis factor-alpha; IL-6 = interleukin-6; AChE = acetylcholinesterase; BChE = butyrylcholinesterase.

## Data Availability

No new data were created or analyzed in this study.
